# Influence of Low Temperature Plasma Oxidizing on the Bioactivity of NiTi Shape Memory Alloy for Medical Applications

**DOI:** 10.3390/ma16186086

**Published:** 2023-09-06

**Authors:** Justyna Witkowska, Tomasz Borowski, Agnieszka Sowińska, Emilia Choińska, Dorota Moszczyńska, Jerzy Morgiel, Jerzy Sobiecki, Tadeusz Wierzchoń

**Affiliations:** 1 Faculty of Materials Science and Engineering, Warsaw University of Technology, 02-507 Warsaw, Poland; tomasz.borowski@pw.edu.pl (T.B.); emilia.choinska@pw.edu.pl (E.C.); dorota.moszczynska@pw.edu.pl (D.M.); jerzy.sobiecki@pw.edu.pl (J.S.); tadeusz.wierzchon@pw.edu.pl (T.W.); 2 Pathology Department, Children’s Memorial Health Institute, 04-730 Warsaw, Poland; a.sowinska@ipczd.pl; 3 Institute of Metallurgy and Materials Science, Polish Academy of Sciences, 30-059 Krakow, Poland; j.morgiel@imim.pl

**Keywords:** NiTi shape memory alloy, glow discharge oxidizing, biocompatibility, bioactivity, osseointegration

## Abstract

The present study elucidates the impact of glow discharge oxidation within a low-temperature plasma environment on the bioactivity characteristics of an NiTi shape memory alloy. The properties of the produced surface layers, such as structure (TEM observations), surface morphology (SEM observations), chemical and phase composition (EDS and XRD measurements), wettability (optical gonimeter), and the biological response of osteoblasts and platelets to the oxidized surface compared with the NiTi alloy without a surface layer are presented. The presented surface modification of the NiTi shape memory alloy, achieved through oxidizing in a low-temperature plasma environment, led to the creation of a continuous surface layer composed of nanocrystalline titanium oxide TiO_2_ (rutile). The findings obtained from this study provide evidence that the oxidized layer augments the bioactivity of the shape memory alloy. This augmentation was substantiated through the spontaneous biomimetic deposition of apatite from a simulated body fluid (SBF) solution. Furthermore, the modified surface exhibited improved osteoblast proliferation, and enhanced platelet adhesion and activation. This proposed surface modification strategy holds promise as a prospective solution to enhance the biocompatibility and bioactivity of NiTi shape memory alloy intended for prolonged use in bone implant applications.

## 1. Introduction

NiTi alloy, with a chemical composition close to equiatomic, is categorized as a smart material and has growing prominence within the medical field. This prominence is due to its distinctive attributes, notably shape memory and superelasticity [1,2]. These properties enable the design of innovative bone or cardiac implants and medical instruments [3,4,5]. The biocompatibility of NiTi alloys is held in high regard, primarily thanks to a self-passivation mechanism wherein a thin and enduring oxide layer spontaneously forms on the alloy’s surface, which is related to the strong chemical affinity of titanium to oxygen [6]. It is the passive layers that are responsible for the corrosion resistance of NiTi alloy, which is comparable [7] or even higher [6] than that of stainless steels. However, as indicated in the literature, the passive layers formed on the NiTi alloy are thin (5–8 nm) [8], and their rate of self-rebuilding in case of damage may be slower than that of dissolution in the environment of the human body [9,10]. As demonstrated by Toker et al. [11], the efficiency of NiTi alloy surface passivation, and thus the degree of its biocompatibility, also depends on the geometry of the implant and the place of implantation in the body. Damage to the passive layer, e.g., as a result of prolonged exposure to physiological fluids, intensifies the process of releasing Ni ions from the surface [8]. In addition, the spontaneously formed passivated layer may contain small amounts of nickel oxides (NiO and Ni_2_O_3_) and metallic Ni [12], which is released from the surface more easily than in the form of oxides. It should be noted that the rate of nickel release to the surrounding environment decreases over time [13].

Therefore, the main problem in the application of NiTi alloy in medicine still remains its high nickel content (over 50% Ni), which although present in trace amounts in the human body, can cause allergies when the safe daily dose (approx. 200–300 μg) is exceeded, and exhibit cytotoxic and even carcinogenic effects [14,15,16,17,18,19]. Chrzanowski described changes in the osteoblast cytoskeleton after contact with metal ions, including nickel, of various concentrations [19]. He found that even low concentrations (0.1–0.5 mM) of nickel, cobalt and vanadium ions had a negative effect on the biological response of cells, which means that they are more cytotoxic than, for example, aluminum, chromium, molybdenum and iron, of which only a higher concentration (10 mM) translated into changes in the cytoskeleton of cells [19]. In his works [16,17], Plant showed the negative effect of nickel ions on human vascular endothelial cells, in which increased oxidative stress and impaired function were observed. Wataha et al. [20] studied the secretion of inflammatory cytokines by macrophages exposed to various metal ions, including nickel. It was found that although there were no clear changes in the level of inflammatory cytokines (IL-1 β and TNF-α) after 24 h, after 72 h the levels of nickel ions had significantly increased. It is worth noting that in in vitro and in vivo studies, inflammatory reactions appeared during exposure to concentrations of metal ions that were previously released from alloys used in dentistry. Similar observations were also noted during the study of cells exposed directly to NiTi alloy and other alloys used in medicine [21].

In pursuit of improving resistance against corrosion and tailoring the biological characteristics of the NiTi alloy to suit specific applications, various surface engineering methods are employed, such as oxidation in air and steam, electrochemical and laser methods, ion implantation, glow discharge treatments, PVD and CVD methods, and deposition of carbon, ceramic or polymer coatings [22,23,24,25,26,27]. Prospective processes are those that enable the treatment of complexly shaped implants generally constituting bone implants made of shape memory alloys, and that guarantee good adhesion of the surface layers, full control of microstructure, phase composition, surface topography, and thus enable the shaping of such properties as wettability, corrosion resistance and biological properties to ensure their compatibility with the intended use of the implants.

Our prior investigations have demonstrated that enhancing corrosion resistance, biocompatibility, and diminishing the release of nickel ions into the biological milieu can be accomplished through the application of low-temperature plasma treatment techniques under glow discharge conditions [22,23,24,25,26,27]. Nonetheless, the chemical bioactivity of layers produced during glow discharge processes, denoting the capacity to deposit calcium phosphates, specifically hydroxyapatite, in a biomimetic manner, remains inadequately understood [28]. This facet assumes great significance in the potential utilization of shape memory alloys for fabricating long-term bone implants as heightened bioactivity correlates with expedited osseointegration of the implant with the patient’s bone structure. An example of such a potential application is the TKR (total knee replacement) knee implant. The choice of an optimal metal alloy for the femoral and tibial parts of the implant plays a significant role in prolonging the life of TKR implants and preventing the need for revision surgery. Recently, a multiple-criteria decision-making analysis (MCDM) [29] has been used to help find a suitable TKR implant material, and it has been shown that NiTi shape memory alloys are the most prospective material for this purpose (porous NiTi alloy was ranked first, followed by solid NiTi alloy). Currently used materials, such as stainless steels, CoCr alloys, or other titanium alloys were ranked lower [30]. However, the slow process of osteogenesis, as well as the production of fibrous tissue at the interface of NiTi alloy implants with the bone, leading to poor bonding of the prosthesis with the surrounding bone and to micro-movements of the implant, make it necessary to use surface modifications that increase its bioactivity. Hence, the production of thin apatite layers on NiTi alloy implants are seen as the solution to increasing and accelerating osseointegration [31]. However, due to the porous structure of apatite layers and their poor adhesion to the substrate [32], they alone do not provide sufficient protection against metallosis. This could be ensured, for example, by prior oxidation of the alloy.

In light of the above, this work aims to investigate the impact of the glow discharge oxidizing process in low-temperature plasma on the bioactive properties of a shape memory alloy. The assessment is performed via incubation in SBF (simulated body fluid) solution and examination of the resulting apatite layers. A method of producing layers at temperatures up to 300 °C is presented, which guarantees the preservation of the specific properties of NiTi alloy, i.e., the effect of shape memory and superelasticity [33]. In addition, the properties of this layer, such as structure, morphology, chemical composition or wettability, as well as the analysis of the biological reactions elicited from osteoblasts and platelets in response to the oxidized surface, in relation to the NiTi alloy in its initial state are also presented. The results of the study can be used as the basis for the development of new material solution concepts for bone implants with shape memory for long-term use.

## 2. Materials and Methods

The researched material consisted of an NiTi shape memory alloy with a chemical composition of 50.8 at.% Ni (Ti—rest). Disc-shaped samples with a diameter of ø8 mm and a thickness of 1 mm were used in the tests. The samples were mechanically ground with up to 1200-grit sandpaper and degreased in acetone. The glow discharge oxidizing process was conducted at a temperature of 290 °C for 60 min in oxygen plasma at a chamber operating pressure of 1.6 mbar. The temperature was controlled by a thermocouple. Before oxidizing, the specimens were heated to 290 °C in nitrogen plasma (N_2_ atmosphere with 99.999% purity).

NiTi samples in the initial state and with an oxidized surface layer were incubated in SFB solution prepared according to Kokubo [34]. The composition is given in the table below (Table 1). The samples were incubated for 14 and 30 days in an incubator at 37 °C. The solution was changed every 5 days.

The microstructure investigations were carried out using a TECNAI SuperTWIN (200 kV) FEG transmission electron microscope (TEM) (FEI, Eindhoven, The Netherlands) capable of working in scanning mode (STEM). The nanoscale local chemical analysis was performed with an integrated EDAX energy dispersive microanalysis (EDS) system (EDAX, Tilburg, The Netherlands).

The surface morphology of the samples before and after incubation in SBF solution was imaged using a scanning electron microscope (Hitachi, Tokio, Japan, S-3500N) with an acceleration voltage of 15 keV in SE (secondary electron) mode. An EDS (Energy-dispersive X-ray spectroscopy) attachment was used for analysis of the chemical composition of the biomimetic apatite layers on the sample surface after incubation.

The XRD measurements were performed on a Bruker (Billerica, MA, USA) D8 Advance diffractometer, equipped with parallel beam optics and a Cu Kα radiation (λ = 0.154056 nm) source that operated at 40 kV and 40 mA at room temperature. The scan optics consisted of a Göbel mirror on the side of the incident beam and twin secondary Soller slits in the diffracted beam. The measurements were performed with a fixed angle of incidence of the primary beam equal to 3°, over the 2θ range, between 20° and 80°, with a step size of 0.025° and a 3 s counting time per step.

The wettability test was performed with a goniometer—Contact Angle System OCA 20 (DataPhysics Instruments, Filderstadt, Germany). Measurements were made at room temperature for two liquids: distilled water and diiodomethane. After applying a drop of 0.4 μL to the surface of each sample its image was recorded immediately after stabilization. Average values and standard deviations of the contact angles were calculated from 5 measurements performed for each sample. The profiles of the droplets were analyzed using dedicated SCA20 software. Based on the obtained values of contact angles for distilled water and diiodomethane, the surface free energy was calculated for each sample using the standard Owens–Wendt method [35].

Studies of osteoblast proliferation on the titanium oxide layers produced and the NiTi alloy were conducted with the use of Normal Human Osteoblasts (NHOst, Lonza, Basel, Switzerland). The cells were grown in an osteoblast basal medium enriched with fetal bovine serum (50 mL/500 mL medium), ascorbic acid (0.5 mL/500 mL medium), GA-1000 (0.5 mL/500 mL medium) antibiotics (OGM Osteoblast Growtth Medium BulletKit, Lonza), in a humid atmosphere of 95% air and 5% CO_2_ at 37 °C. The medium was changed every 48 h. Osteoblasts suspended in the incubation medium at a concentration of 3 × 10^4^/500 μL were applied onto NiTi samples in the initial state and after glow discharge oxidizing. The cells were then incubated for 24, 48 h and 6 days at 37 °C. Following incubation, the culture was rinsed with a phosphate buffered saline solution (PBS, Lonza), and the settled cells were fixed in 4% glutaraldehyde for 30 min at 4 °C before rinsing in a cacodylic buffer, in accordance with the procedure for preparing biological material for examinations employing a scanning electron microscope. The prepared specimens were vacuum-coated with a ca. 10–15 nm layer of gold using a JFC-1200 JEOL fine coater. They were then tested under a scanning electron microscope (JSM-7600F, JEOL, Tokyo, Japan) with an acceleration voltage of 5 keV in LEI (lower secondary electron image) mode.

Adhesion and aggregation tests of blood platelets on the surface of NiTi alloy in the initial state and after producing an oxidized layer were performed with the use of platelet rich plasma (PRP), prepared using the blood of healthy donors. The plasma was incubated on the surface of the samples for 2 h at 37 °C. After this time, the non-adhering cells were rinsed off, while the adhering ones were fixed in 4% glutaraldehyde for 30 min at 4 °C, rinsed in a cacodylic buffer and dehydrated and observed under a scanning electron microscope according to the same procedure used for the osteoblast cultures.

The number of osteoblasts, as well as platelets and their aggregates, was measured using morphometric CellSens (Olympus, Hamburg, Germany) software. The results are presented as means and standard deviations. Statistical analyses were performed using the Fisher and Student’s t-tests at a significance level of α = 0.05.

## 3. Results

**Figure 1:**
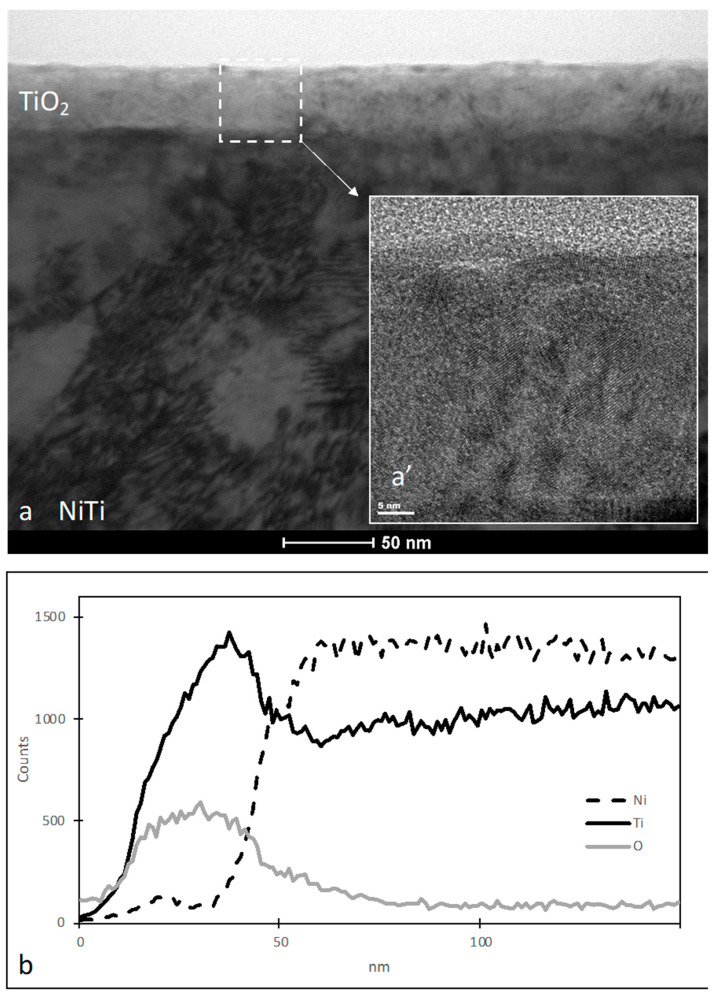
Microstructure of TiO_2_ layer produced on NiTi alloy (**a**); HRSTEM image of TiO_2_ layer (**a’**); and linear distribution of elements in the TiO_2_ layer (**b**).

Images from the transmission electron microscope (TEM) show the cross-section of the oxidized surface layer produced at low-temperature plasma on the NiTi shape memory alloy (Figure 1a). As we know from our earlier studies [23], this layer consists of titanium dioxide: TiO_2_ rutile-type with a nanocrystalline structure. The thickness of the titanium oxide layer is approx. 40 nm. The chemical composition of the layer is also confirmed by the linear distribution of elements obtained from EDS (Figure 1b).

**Figure 2:**
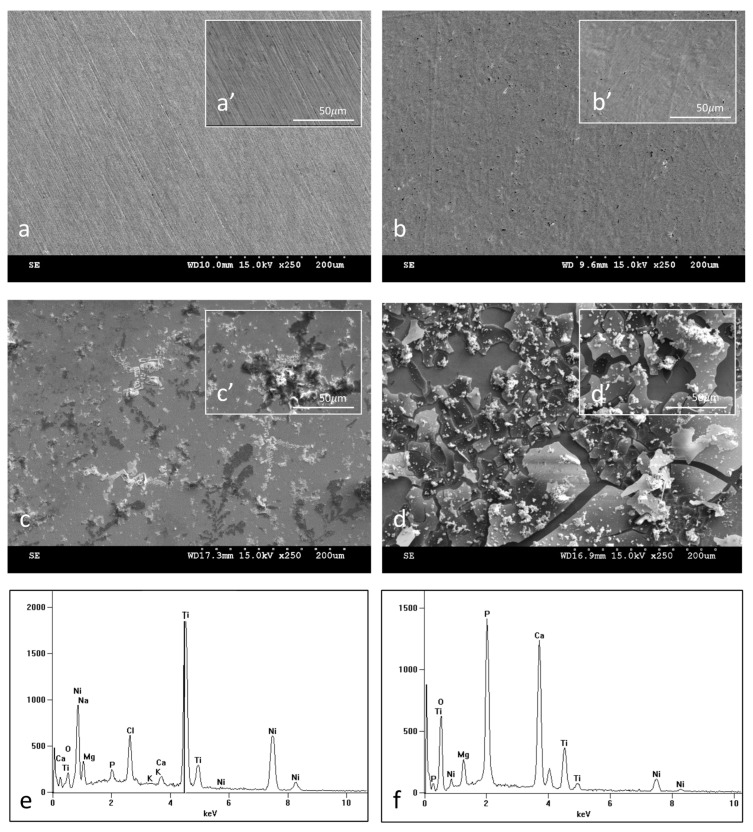
Surface morphology of NiTi in initial state (**a**,**a’**,**c**,**c’**) and with a TiO_2_ surface layer (**b**,**b’**,**d**,**d’**) both before (**a**,**a’**,**b**,**b’**) and after (**c**,**c’**,**d**,**d’**) 14-day incubation in SBF solution: SEM images magnified ×250 (**a**–**d**), ×1000 (**a’**–**d’**). Chemical composition obtained by EDS from the entire image area for NiTi non-oxidized (**e**) and with a TiO_2_ surface layer (**f**) after 14-day incubation in SBF solution.

**Figure 3:**
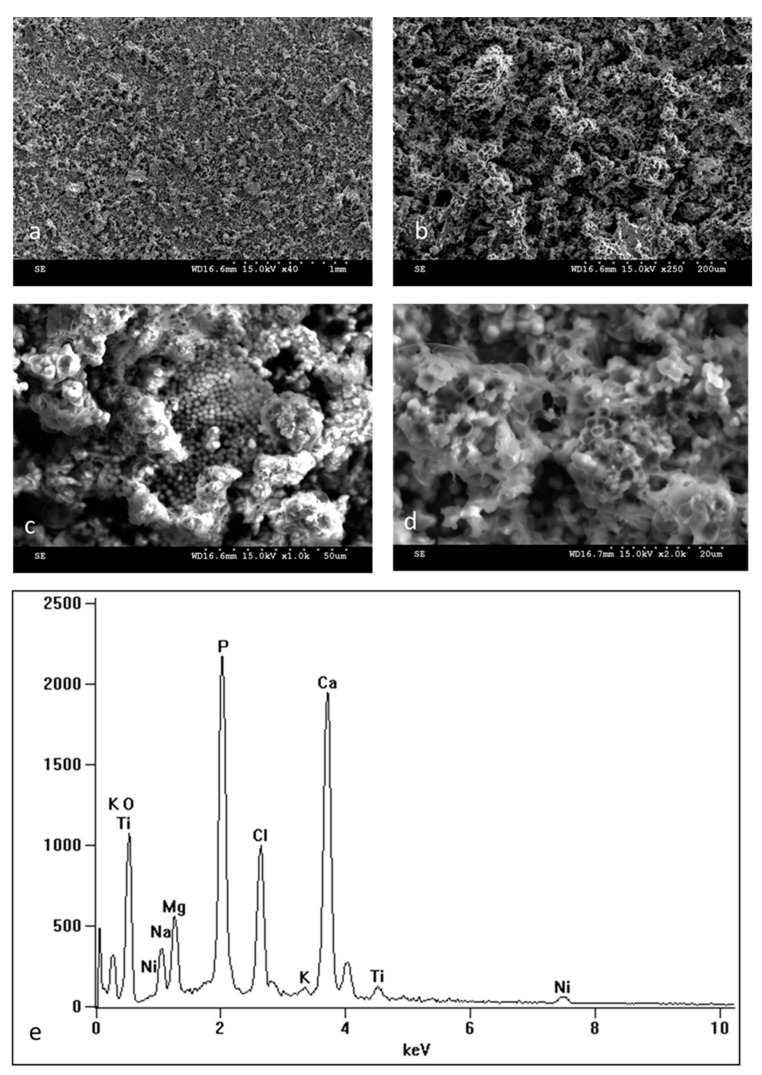
Surface morphology of calcium phosphates biomimetically deposited on NiTi alloy with a TiO_2_ surface layer after 30 days of incubation in SBF: SEM images magnified ×40 (**a**), ×250 (**b**), ×1000 (**c**), ×2000 (**d**). Chemical composition obtained by EDS from the entire image area for NiTi with a TiO_2_ surface layer after 30-day incubation in SBF solution (**e**).

Scanning electron microscope (SEM) observations show the surface morphology of NiTi samples in the initial state (Figure 2a,a’,c,c’) and with an oxidized surface layer (Figure 2b,b’,d,d’) both before (Figure 2a,a’,b,b’) and after (Figure 2c,c’,d,d’) 14 days of incubation in SBF. The surface topography of the oxidized layer before incubation reflects the surface of the initial samples which include delicate scratches resulting from grinding the samples. Observations of the samples after 14 days of incubation (Figure 2c,c’,d,d’) show numerous deposits formed in the biomimetic process of deposition of apatite layers as a result of the interaction of the material surface with the ions present in the SBF solution. It is clearly visible from the SEM images that apatite deposits are formed more easily on the surface of the samples with the oxidized layer (Figure 2d,d’), and there are many more of them after the same incubation time than on the non-oxidized samples (Figure 2c,c’). The EDS analysis confirmed the predicted chemical composition of apatite deposits, which according to the spectra presenting the elemental analysis of the whole image (Figure 2e,f), consist of, among others, calcium, phosphorus, sodium, magnesium, potassium, oxygen etc. The other peaks that belong to titanium and nickel come from the substrate and the surface layer. The amount of calcium and phosphorus in the elemental composition of the tested surfaces is higher for samples with an oxidized layer than for non-oxidized samples (Table 2). Both the images of the formed deposits and their chemical composition testify to the increased bioactivity of the samples with the oxidized layer. To confirm the bioactivity of the prepared surface layer, the incubation process of the samples with the TiO_2_ layer was extended to 30 days. Surface images (Figure 3a–d) as well as the EDS spectra from the entire image area (Figure 3e) after 30 days of incubation clearly show a solid apatite layer of calcium phosphates that has been formed on the surface, which further confirms the excellent bioactivity of this layer. Also, a point analysis of apatite deposits for samples soaked for 14 and 30 days confirms the formation of calcium phosphates (Table 2). The ratio of Ca to P ranges from 1.26 to 1.53 and is the highest for samples with an oxidized layer soaked for 30 days. It is worth noting that this ratio is close to that found in ideal hydroxyapatite (1.67), which indicates the formation of calcium phosphates that can potentially promote better osseointegration of the material.

**Figure 4:**
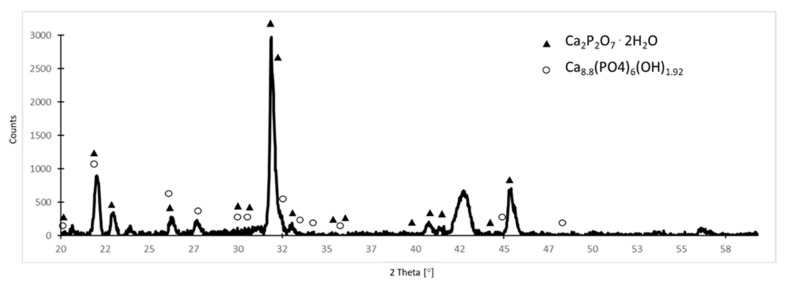
XRD pattern for NiTi with a TiO_2_ surface layer after 30-day incubation in SBF solution: marked peaks for identified calcium phosphates.

Usually, the individual components of different types of calcium phosphate compositions are mixed together and strongly integrated with each other, and therefore, the X-ray diffraction technique is very helpful in identifying phases [36]. XRD analysis of the samples after 30 days of incubation in the SBF solution showed that on the surface of NiTi with a TiO_2_ surface layer, a multiphasic composition of calcium phosphates consisting of calcium pyrophosphate dihydrate Ca_2_P_2_O_7_·2H_2_O (known as CPPD) and hydroxyapatite was formed (Figure 4). Datasheets indicate that it is a non-stoichiometric calcium-deficient hydroxyapatite Ca_8.8_(PO_4_)_6_(OH)_1.92_ which is often found with biomimetic compounds formed naturally when samples are soaked in solutions resembling human body fluids and is referred to as bone-like apatite. This would explain the observation that although the EDS analysis shows much more Ca and P after 14 days of incubation on samples with the oxide surface layer than on the non-oxidized samples, the Ca/P ratio is lower, indicating more CDHAP (calcium-deficient hydroxyapatite) produced, which is in line with the SEM observations.

The contact angle of NiTi samples increased after low-temperature oxidizing, which means decreased wettability for both polar (distilled water) and non-polar liquids (diiodomethane) (Table 3). Considering the classification based on 90 degrees as the critical point, it can be stated that the surface changed its character from slightly hydrophilic to slightly hydrophobic. The results were used to calculate the surface free energy, which decreased after the oxidation process (Table 3).

**Figure 5:**
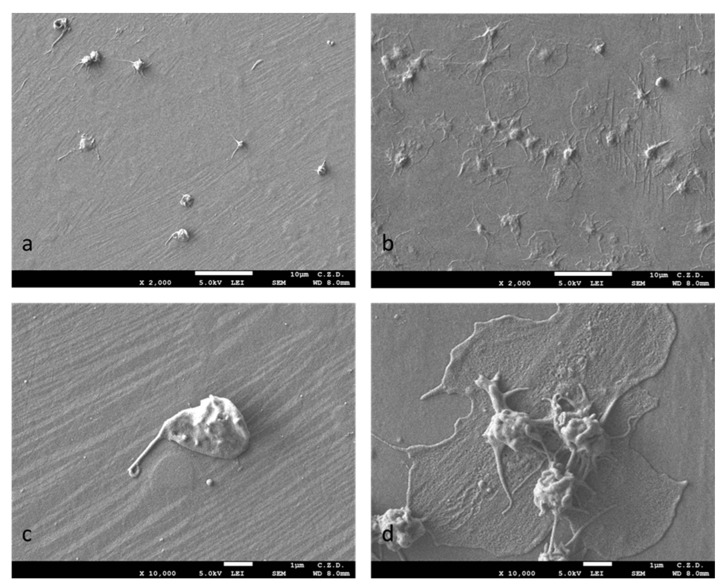
Morphology of platelets adhering on the surfaces of NiTi alloy in the initial state (**a**,**c**) and with a TiO_2_ surface layer (**b**,**d**). SEM images magnified ×2000 (**a**,**b**) and ×10,000 (**c**,**d**).

**Figure 6:**
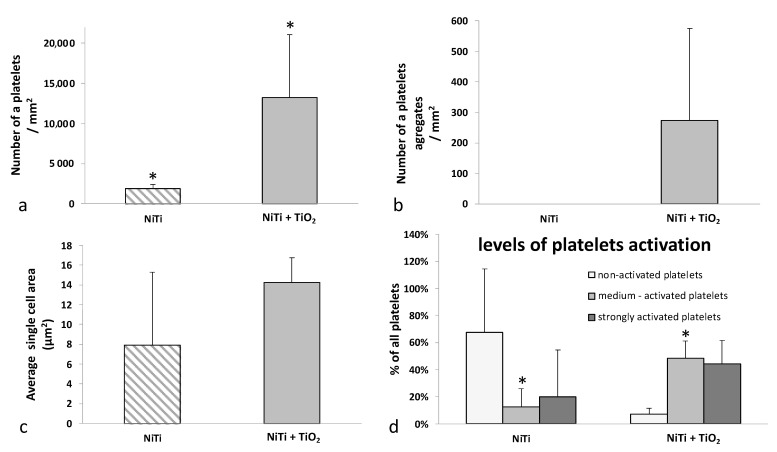
Quantitative assessment of platelet adhesion (**a**), aggregation (**b**), their size (**c**) and level of activation according to morphological features (**d**) for NiTi in initial state and with a TiO_2_ surface layer; * *p* ≤ 0.05 NiTi versus NiTi + TiO_2_.

Platelet adhesion studies showed statistically significant differences in cell adhesion for samples with a TiO_2_ oxidized layer compared with the non-oxidized NiTi samples. The number of thrombocytes was much higher for samples with oxidized layers, and furthermore, they formed numerous aggregates, which was revealed both by microscopic observations of adhered cells using a scanning electron microscope (SEM) (Figure 5a–d) and by measurements made using morphometric software, as presented in graph form in Figure 6a–d. No platelet aggregation was observed for the samples without the surface layer, and the average size of a single cell was almost half that of the samples with the oxidized layer. Differences in aggregation and cell size are clearly noticeable but not statistically significant.

Platelet activation was assessed on the basis of their morphology according to the classification described in the literature [37]. The platelets were divided into “non-activated” with a spherical shape, “medium-activated”, i.e., those with numerous dendritic protrusions, and “strongly activated”—the most flattened. On both types of surface (with and without an oxidized surface layer) cells with different degrees of activation were found, with the majority of cells observed in the non-oxidized samples being non-activated cells, and strongly activated cells being moderately represented in the samples with the oxidized layer (Figure 6d).

**Figure 7:**
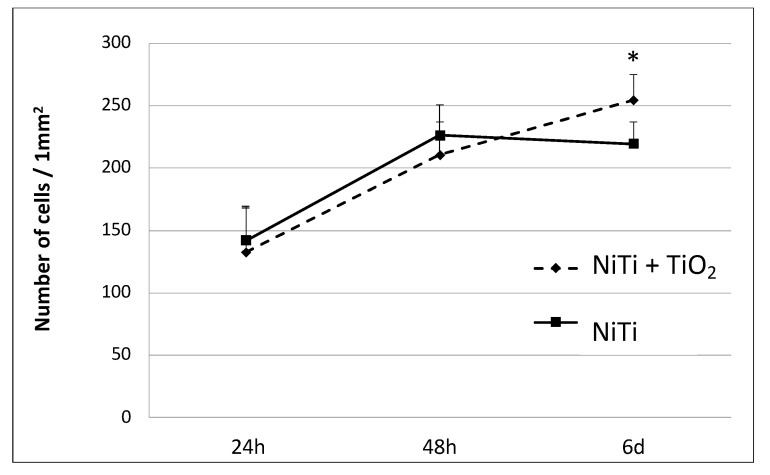
Proliferation of osteoblasts cultured on NiTi in the initial state and with a TiO_2_ surface layer represented as the number of cells per mm^2^ and measured after 24 h, 48 h and 6 days of growth; * *p* ≤ 0.05 NiTi versus NiTi + TiO_2_.

Osteoblast proliferation was preserved on both sample types and within the first 48 h they proliferated at a similar rate. Between days 2 and 6, osteoblast growth was inhibited on the samples in the initial state, while on samples with an oxidized layer, they continued to proliferate at only a slightly reduced rate, as evidenced by the slope of the curve in the graph (Figure 7). As a result, after 6 days of incubation, the number of osteoblast cells was significantly higher on the surface of the samples with the oxidized layer compared with the initial samples.

**Figure 8:**
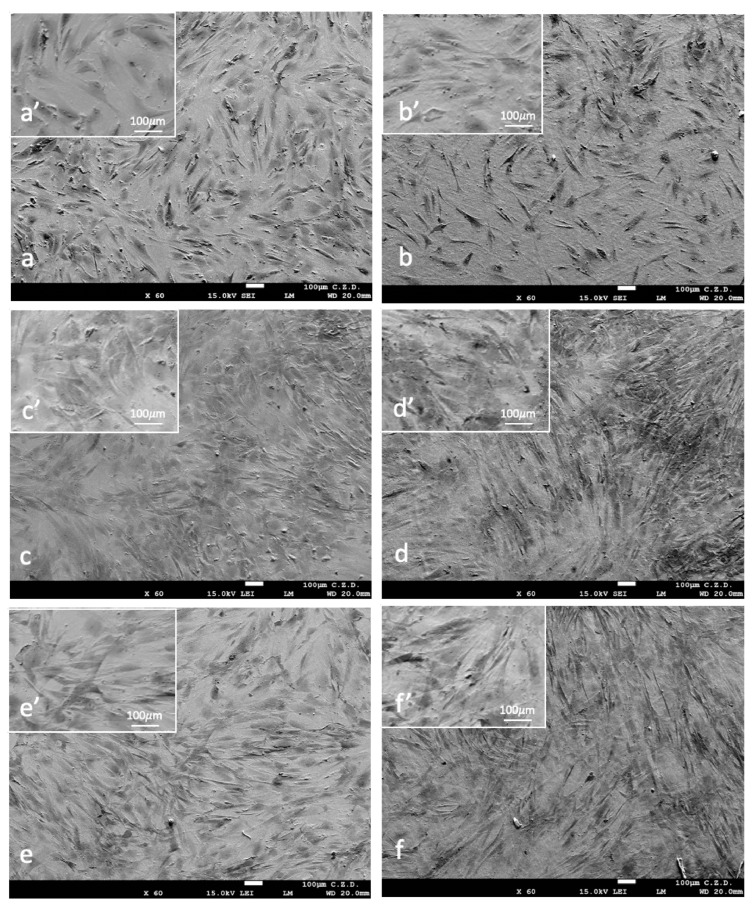
Morphology of osteoblasts cultured on the surfaces of NiTi alloy in the initial state (**a**,**a’**,**c**,**c’**,**e**,**e’**) and with a TiO_2_ surface layer (**b**,**b’**,**d**,**d’**,**f**,**f’**) for 24 h (**a**,**a’**,**b**,**b’**), 48 h (**c**,**c’**,**d**,**d’**) and 6 days (**e**,**e’**,**f**,**f’**). SEM images magnified ×60 (**a**–**f**), ×500 (**a’**–**f’**).

The morphology of osteoblasts growing on samples with and without an oxidized surface layer was normal and slightly changed during the experiment (Figure 8). After 24 h (Figure 8a,a’,b,b’) and 48 h (Figure 8c,c’,d,d’) of incubation, the population of osteoblasts was heterogeneous, i.e., some of the cells had a flattened shape, and some were oblong or spindle-shaped (Figure 8a,a’,b,b’). At the end of the experiment (Figure 8e,e’,f,f’), most of the cells were oblong. After a 6-day incubation period, osteoblasts evenly covered the surface of both types of samples and overlapped each other.

## 4. Discussion

The NiTi shape memory alloy, being self-passivating, is considered a material with good biocompatibility [38,39,40]. However, surface modifications are necessary in the context of medical applications [39,41]. A large amount of literature on the use of various surface engineering methods to modify NiTi alloy [8,17,22,24,26,33,39,40,41,42,43,44,45,46,47,48,49,50,51,52,53,54,55,56,57,58] indicates that improving its biocompatibility and bioactivity is an important and current problem, especially in the case of long-term implants.

The produced surface layers were characterized by a nanocrystalline structure of titanium oxide TiO_2_ (rutile), which is the most thermodynamically stable polymorphic variety of titanium oxide [6]. The main motivations for studying the bioactivity of this layer type were the results of our previous studies, which indicated that the layers produced under glow discharge conditions increase corrosion resistance, thereby providing good protection against the release of nickel ions into the biological environment [22,24]. Moreover, in our preliminary studies [27], it was shown that the TiO_2_ layer of titanium oxide increases adhesion to the NiTi substrate. Those studies investigated amorphous carbon coating deposition. Our current study shows that the TiO_2_ layer also ensures faster surface deposition of an apatite layer composed of calcium phosphates.

The use of a glow discharge process temperature below 300 °C made it possible to produce surface layers that retain specific properties of the NiTi alloy, i.e., shape memory and superelasticity, which was previously demonstrated [33,59] on the samples of nitrided (TiN) or oxynitrided (TiO_2_ + TiN) layers with a thickness of several dozen nanometers and produced on NiTi alloy at different temperatures. It was found that during glow discharge processes carried out at temperatures above 300 °C, Ni_4_Ti_3_ phase precipitates appear in the structure of the alloy [33]. These processes can also form an Ti_2_N sublayer [60], as well change the kinetics of martensitic transformation [61] (appearance of an additional rhombendric R phase between the transition from austenitic to martensitic phase). It has been observed that processes carried out at temperatures below 300 °C do not adversely affect the shape memory phenomenon, and may even have a positive effect on the superelastic properties of NiTi alloy, expanding the hysteresis loop in the stress–strain relationship [61]. Moreover, nitrided and oxynitrided layers with a nanocrystalline structure that is several tens of nanometer thick did not show any cracks or damage after shape recovery tests [61], which is mainly due to their nanocrystalline structure and low thickness.

Bioactivity, defined as the ability to form a biomimetic bone-like apatite layer in the environment of solutions simulating human body fluids, is especially important in the context of the long-term use implants. There are numerous studies confirming that in vitro studies utilizing the biomimetic method also produce results in vivo, as well as a palpably better connection between the bioactively enhanced material and the bone [34]. This is based on the assumption that the biomimetic apatite layer, which is formed more easily on more bioactive surfaces, resembles biological bone crystals, and therefore, new bone formation can occur more readily on the surface [62]. Many types of calcium phosphates are used and synthetically produced as materials or layers for orthopedic or dental applications [36]. Of these, the most common and also the most stable is hydroxyapatite (HA). However, biologically occurring HA differs from synthetically produced HA, which has a Ca to P ratio of 1.67. In biological systems, calcium-deficient hydroxyapatite (CDHA, with the formula Ca_10−x_(HPO_4_)_x_(PO_4_)_6−x_ (OH)_2−x_ is found, most often also containing other ions (Na, K, Mg, Cl, etc.). In this respect, biomimetic CDHA is more like an inorganic composition of natural bone, which also shows a lower Ca/P ratio than synthetic bone. Therefore, CDHA, which has a higher specific surface area than HA or tricalcium phosphate (TCP) [63], exhibits considerable promise for industrial production of synthetic bone substitutes [64], with potential applications extending to drug delivery systems. Its presence in the biomimetically deposited layer ensures good biocompatibility and bioactivity of oxidized NiTi. According to the various in vitro experiments available in the literature, HA or CDHA is not precipitated directly from solutions containing calcium and orthophosphate ions, but some phases are present earlier as precursors [65,66,67,68], for example, dicalcium phosphate dihydrate (DCPD, brushite), amorphous calcium phosphate (ACP) or octacalcium phosphate (OCP) [36]. They are also discussed as precursors of apatite formation in vivo. Many researchers also use concentrated SBF solutions with higher ion concentrations (e.g., 1.5 SBF) to speed up in vitro deposition observations [69].

In addition to the observed deposition of CDHA, calcium pyrophosphate dihydrate Ca_2_P_2_O_7_·2H_2_O (CPPD) was also found in the apatite layers deposited on the samples, which although less popular, has recently been intensively studied as a material of biological interest [70]. The research included, for example, the production of ceramics based on calcium pyrophosphate for potential osteoplastic applications [71], as well as the use of calcium pyrophosphate for localized drug delivery in dental applications, resulting in a strong antibacterial effect towards *E. coli* (74% growth inhibition) and *S. aureus* (48% growth inhibition) [72]. It was also used for fabrication of a new composite material based on β-calcium pyrophosphate and Ti-13Nb-13Zr, which resulted in higher bioactivity compared with the pure Ti-13Nb-13Zr alloy [73].

Better proliferation of such cells as osteoblasts, mesenchymal cells, or platelet adhesion in vitro suggest higher in vivo bioactivity. It is known that platelets contain growth factors, which ensure better healing as well as greater growth of various types of cells. PRP is used as a recovery agent for musculoskeletal injuries. The results presented in this paper are consistent with these observations since the material with the oxidized layer was characterized by both increased proliferation of osteoblasts, and platelet adhesion and aggregation. In our study, osteoblast proliferation was similar for the first 48 h for both types of material. Only after 6 days of proliferation can a statistically significant difference be seen in favor of samples with a surface layer. This is due to the greater biocompatibility of the titanium oxide surface and probably the absence of the cytotoxic effect of nickel. This is consistent with studies from the literature [15,20] which indicate that sometimes the negative effect of nickel appears after 72 h, but is not observed after only 24 h. In addition, the layer of calcium phosphates indicates the formation of compounds beneficial for accelerated osseointegration. It can, therefore, be concluded that the presented modification of the shape memory alloy surface allows for a significant improvement in bioactivity. It is, therefore, advantageous in terms of increasing the biocompatibility of the material for potential applications as bone implants.

The response of cells, as well as the bioactivity of the surface, is certainly influenced by many factors, one of them undoubtedly being the wettability of the surface. The findings of this study align with previous literature findings suggesting that surfaces with lower surface energy and increased hydrophobicity promote platelet adhesion [74,75,76,77,78,79,80,81] or proliferation of other cell types. Many studies propose that this phenomenon may be attributed to the enhanced adsorption of proteins, which facilitate cell adhesion, on hydrophobic surfaces compared with hydrophilic surfaces where water molecules strongly adhere and hinder protein adsorption [82,83]. However, conflicting reports can also be found in the literature, indicating that hydrophobic surfaces may inhibit adhesion or cell growth on the surface [8,84,85,86,87], while other studies claim there is no significant effect caused by differences in contact angles on the biological response of cells (e.g., platelets [88]) on the surface of the tested biomaterials.

The chemical composition, phase composition, and surface wettability are important, yet are not the sole factors determining material surface interaction with the biological environment. Surface topography and surface charge are additional significant factors that can influence cell adhesion [89,90] but were not specifically investigated in this work. Future research should delve into these aspects and also examine the composition and distribution of the protein biofilm on the sample surfaces. It is well established that the types of proteins present and their relative proportions play a role in determining cell adhesion outcomes [88].

## 5. Conclusions

In this study, we have introduced a surface modification approach for the NiTi shape memory alloy. This modification involves oxidation conducted under glow-discharge conditions in a low-temperature plasma environment, yielding a continuous nanometer-thick layer of nanocrystalline titanium oxide TiO_2_ (rutile). The low temperature in this process ensures the preservation of the alloy’s specific properties inherent to shape memory. Our research has demonstrated that this newly formed oxide layer significantly heightens the bioactivity of the NiTi shape memory alloy. This was substantiated by the rapid and spontaneous biomimetic deposition of apatite from a simulated body fluid (SBF), a phenomenon that occurred more expeditiously on the modified surface compared with the unmodified material. Additionally, our investigations revealed enhanced osteoblast proliferation on the modified surface, as well as increased adhesion and activation of platelets obtained from platelet-rich plasma (PRP). Furthermore, the presence of hydroxyapatite among the calcium phosphates within the biomimetic layer formed in vitro holds promise for improved in vivo bioactivity. This optimism is derived from the fact that the SBF solution utilized closely mimics both the composition and pH of human body fluids. Consequently, our proposed surface modification presents a prospective avenue for enhancing the biocompatibility of NiTi alloy employed in the development of long-term bone implants.

## Figures and Tables

**Table 1:** Composition of SBF according to Kokubo [34].

Component	Concentration (g/L)
NaCl	8.035
NaHCO_3_	0.355
KCl	0.225
K_2_HPO_4_·3H_2_O	0.231
MgCl_2_·6H_2_O	0.311
CaCl_2_	0.292
Na_2_SO_4_	0.072
(HOCH₂)₃CNH₂(Tris)	6.118

**Table 2:** The amount of calcium, phosphorus and oxygen expressed in atomic % determined by EDS measurements for non-oxidized NiTi after 14 days of SBF incubation and NiTi with a TiO_2_ oxidized surface layer after 14 and 30 days of SBF incubation. Results obtained for measurements from the image area and the average of 3 point measurements.

	Ca (%at.)		P (%at.)		O (%at.)		Ca/P	
Sample	Image Area	Point	Image Area	Point	Image Area	Point	Image Area	Point
NiTi (after 14 days in SBF)	1.72	5.57 ± 0.13	1.32	3.78 ± 0.13	7.55	11.16 ± 0.35	1.30	1.47
NiTi + TiO_2_ (after 14 days in SBF)	19.77	21.70 ± 0.20	14.92	17.22 ± 0.11	48.00	55.34 ± 0.55	1.32	1.26
NiTi + TiO_2_ (after 30 days in SBF)	20.49	24.83 ± 0.18	14.72	16.19 ± 0.13	47.39	33.82 ± 0.73	1.39	1.53

**Table 3:** Contact angles measured for NiTi in the initial state and with a TiO_2_ surface layer using distilled water and diiodomethane, and values of surface free energy calculated for NiTi in the initial state and with a TiO_2_ surface layer.

		NiTi	NiTi + TiO_2_
Contact Angle	distilled water	84.5 ± 4.3	107.1 ± 0.7
	diiodomethane	45.5 ± 1.3	66.5 ± 0.9
Surface Free Energy	γ (mN/m)	36.37	25.78
	γ_d_ (mN/m)	33.7	25.9
	γ_p_ (mN/m)	2.8	0.00

Abbreviations: γ: Total surface free energy; γ_d_: Dispersive component of surface free energy; γ_p_: Polar component of surface free energy.

## Data Availability

Not applicable.
